# Supplementary material for: Methylation-to-Expression Feature Models of Breast Cancer Accurately Predict Overall Survival, Distant-Recurrence Free Survival, and Pathologic Complete Response in Multiple Cohorts
Source: Sci Rep. 2018 Mar 26;8:5190. doi: 10.1038/s41598-018-23494-0 (PMC5979962; doi:10.1038/s41598-018-23494-0)
Supplement: Supplementary file 1 — Supplementary Material [file 41598_2018_23494_MOESM1_ESM.pdf]

Methylation-to-Expression Feature Models of Breast Cancer Accurately Predict Overall Survival, Distant-Recurrence Free Survival, and Pathologic Complete Response in Multiple Cohorts

Jeffrey A. Thompson[1*], Brock C. Christensen[2], and Carmen J. Marsit[3]

[1] Department of Biostatistics, University of Kansas Medical Center
[2] Department of Epidemiology, Geisel School of Medicine at Dartmouth College
[3] Department of Environmental Health, Rollins School of Public Health at Emory University
[*] Corresponding Author:
Jeffrey A. Thompson, Ph.D.
University of Kansas Medical Center
3901 Rainbow Boulevard
5028 Robinson Hall
Kanas City, KS 66160
Phone: (913)-588-8970
jthompson21@kumc.edu

Supplemental Methods

The M2EFM method consists of five primary steps:

1. **Identify differentially methylated loci.** The M2EFM model requires a list of differentially methylated loci between tumor and normal tissue. This can be identified in a discovery dataset using the empirical Bayes method from the *limma* package (1) for R, or a list of known differentially methylated loci can be provided. In this case, we opted to do the latter, thanks to the availability of a list of probes differentially methylated between normal tissue and tumor-adjacent normal tissue that were even more strongly differentially methylated between normal tissue and cancer (2). The 550 most significant CpG loci for differential methylation were passed on to the next step in the process (the exact number of loci to include is not that important but can be determined through cross-validation).
2. **Identify methylation-to-expression quantitative trait loci (m2eQTLs).** m2eQTL analysis involves associating methylation levels at the loci identified in the previous step with gene expression levels genome-wide. In terms of an eQTL analysis, the proportion of methylated alleles for a particular locus is equivalent to the genotype at a single nucleotide polymorphism (SNP), although it is a continuous, rather than discrete value. Identification of m2eQTLs was performed using the *MatrixEQTL* package (3) for R, which builds linear models to test association in a computationally efficient manner. In this way, the M-value of probes in the training data that were found to be differentially methylated in the first step were tested for their association with gene expression patterns in both *cis* and *trans* in a manner analogous to that used in typical eQTL analysis. An m2eQTL was defined to act in *cis* if it was associated with a gene within 10000bp, otherwise it was defined to act in *trans*. The top 110 *trans*-m2eGenes (by effect size, after filtering non-statistically significant results) were passed to the next step, as well as all *trans*-m2eQTLs. For expression-only models all *cis*-m2eGenes that were also involved in *trans*-m2eQTLs were also used (to replace DNA methylation values). This is an update to our previous approach, which used all top *cis* and *trans*-m2eGenes.
3. **Build integrated models of overall survival and distant-recurrence free survival from m2eQTLs and m2eGenes.** The top candidates from the previous step are used to build a joint regression model across both probes and genes involved in the *trans*-m2eQTLs. To cope with the inevitable collinearity of these data and prevent overfitting we used Cox regression with Ridge penalty (4). A molecular risk score was generated for all training samples, by using the exponential function of the weighted sum of the model features.
4. **Integrate clinical variables.** A second regression is used to integrate clinical variables, allowing an easy method of interaction testing between the molecular risk score and clinical predictors, and ensuring that the clinical variables are not penalized along with the genomic data (the clinical variables are typically more informative individually). This has the further benefit of reducing potential correlation of individual genomic predictors with clinical variables. For this step, we performed a Cox regression on the molecular risk score from the previous step and the values of clinical variables: tumor stage and patient age at diagnosis. This allowed us to generate a risk score for all test and validation data, by using this new linear predictor.
5. **Build integrated model of pathologic complete response from m2eGenes.** As in step 4, the top candidates from step 3 are used to build a pCR model for neoadjuvant taxane-anthracycline chemotherapy in breast cancer patients. In this case, a logistic-Ridge regression

model was built. Although it would be possible to perform meta-dimensional data-integration at this step, our data limited us to using only gene expression values in the model (it is nevertheless multi-stage data-integrated). We defined a patient to be treatment sensitive if they were recorded as having either a pathologic complete response, or a residual cancer burden class of RCB 0/I, as in (5). The probability of pCR from the model was used as a chemosensitivity score.

Data

Pre-processing

TCGA, was used to train and test an OS model for breast cancer, and consisted of gene expression and DNA methylation profiles created by TCGA, using the Illumina HiSeq 2000 sequencing and Illumina Infinium HumanMethylation 450 platforms, respectively. Gene expression values were RSEM normalized read counts that were downloaded from the UCSC Cancer Genomics Browser (15). The DNA methylation data were downloaded from the NCI's GDC legacy archive and were background corrected (16) and functionally normalized (17) using the *minfi* package (18) in R. Beta values were transformed into M-values (19). Probes with detection p-values > .01 were labeled NA (not available) and probes with values missing for greater than 50% of samples were removed. The remaining values were imputed using the k-nearest neighbors method, with k=10, from the *impute* package (20), (21) for R. Furthermore, we removed probes on the X or Y chromosomes, those containing SNPs (22), or with cross-hybridization issues (23). Expression data were TDM normalized, which makes the distributions between microarray and RNA-seq datasets similar, to make them more comparable to the validation datasets (24). These data were then batch corrected with the external validation data, using the ComBat function of the *sva* package for R (an empirical Bayes approach) (25). The genes in the RNA-seq data were filtered to include only those also available in the validation data and to remove those that were not expressed, leaving 10990 genes. The samples are described in Table S1. There are 15 samples in TCGA missing outcomes, which we removed. Among 7 samples missing tumor stage we calculated stage from TNM scores for 4 samples, but removed the remaining 3 samples where this was not possible. Finally, 14 samples listed as “Stage X” were also removed, leaving 1028 samples total. There was no evidence of significant differences in the distribution of staging (stage I-IV) between RNA-seq and DNA-methylation data (χ^2 test, p = 0.47), in the TCGA data.

Terunuma, is used for external validation of the OS model, and contains 61 tumor samples with clinical and survival data, downloaded from ArrayExpress (E-GEOD-39004) (26) These data were assayed on the Affymetrix GeneChip Human Gene 1.0 ST Array. They were background subtracted, normalized, and summarized using the rma function of the *oligo* package for R (27). Given that we are using data from multiple platforms, probe-level data were aggregated to gene annotations as the median expression level for probe sets annotated to a gene. These data were further batch corrected using ComBat in conjunction with the following dataset. A description of the samples is in Table S2. None of these data are missing outcomes, however

two samples were missing age at diagnosis. Therefore, we imputed the age values for these two samples from the other clinical annotations using the *mice* package for R.

Kao, is used as a second external validation dataset for the OS model, and contains 327 tumor samples with survival data, downloaded from ArrayExpress (E-GEOD-20685) (28), and was normalized as above. These data were assayed on the Affymetrix GeneChip Human Genome U133 Plus 2.0. A description of the samples appears in Table S2. None of these data are missing outcomes.

Hatzis1, was used to train and test a model of DRFS and another model of pCR, and includes 306, HER2-negative breast cancer cases with neo-adjuvant treatment by taxane-anthracycline (followed by endocrine therapy for ER-positive cases) (13). These data were assayed on the Affymetrix Human Genome U133A Array. Follow-up for DRFS was conducted for a minimum of three years. They were downloaded from GEO (GSE25055), and were normalized as above and batch corrected in conjunction with the following dataset. A description of the samples appears in Table S3. There were 4 samples missing pCR outcomes that were removed from the analysis.

Hatzis2, was used for external validation of both the DRFS and pCR models of (HER2-negative) breast cancer, and includes 182 HER2-negative breast cancer cases with neo-adjuvant treatment by taxane-anthracycline (followed by endocrine therapy for ER-positive cases) (13). These data were assayed on the Affymetrix Human Genome U133A Array. Follow-up for DRFS was conducted for a minimum of three years. They were downloaded from GEO (GSE25065) and were normalized as above. A description of the samples appears in Table S3. There were 16 samples missing pCR outcomes that were removed from the analysis.

Table S1: Distribution of Samples in TCGA Breast Invasive Carcinoma Data

	RNA-seq Count (%)	*450k Count (%)*	*Overlap Count (%)*
Samples w/ Overall Survival Data	1045	766	743
Stage Missing	7 (0.67)	4 (0.52)	3 (0.40)
Stage I	180 (17.22)	125 (16.32)	122 (16.42)
Stage II	592 (56.65)	427 (55.74)	417 (56.12)
Stage III	235 (22.49)	195 (25.46)	188 (25.30)
Stage IV	17 (1.63)	10 (1.31)	8 (1.08)
Stage X	14 (1.34)	5 (0.65)	5 (0.67)
ER +	577 (55.22)	354 (46.21)	353 (47.51)
ER -	177 (16.94)	109 (14.23)	108 (14.54)
ER Status Indeterminate	2 (0.19)	0	0
ER Status Missing	289 (27.66)	303 (39.56)	282 (37.95)
PR +	505 (48.33)	313 (40.86)	313 (42.01)
PR -	246 (23.54)	147 (19.19)	145 (19.52)
PR Status Indeterminate	4 (0.38)	2 (0.26)	2 (0.27)

PR Status Missing	290 (27.75)	304 (39.67)	283 (38.09)
HER2 +	105 (10.05)	54 (7.05)	54 (7.27)
HER2 -	634 (60.67)	402 (52.48)	401 (53.97)
HER2 Status Equivocal	10 (0.96)	3 (0.39)	3 (0.40)
HER2 Status Missing	296 (28.33)	307 (40.08)	285 (38.36)
PAM50 Basal	138 (13.21)	84 (10.97)	83 (11.17)
PAM50 HER2	64 (6.12)	31 (4.05)	31 (4.17)
PAM50 Luminal A	410 (39.23)	273 (35.64)	272 (36.61)
PAM50 Luminal B	183 (17.51)	123 (16.06)	123 (16.55)
PAM50 Normal-like	22 (2.11)	17 (2.22)	17 (2.29)
PAM50 Missing	228 (21.82)	238 (31.07)	217 (29.21)
Deaths	144 (13.78)	100 (13.05)	95 (12.79)
Mean Age	58.31	58.01	57.99

Table S2: Distribution of Samples in Terunuma and Kao Breast Invasive Carcinoma Data

	Terunuma Count (%)	*Kao Count (%)*
Total Samples	61	327
Stage I	4 (6.56)	68 (20.80)
Stage II	43 (70.49)	147 (44.95)
Stage III	14 (22.95)	104 (31.80)
Stage IV	0	8 (2.45)
ER + [a]	31 (50.82)	200 (61.16)
ER -	30 (49.18)	127 (38.84)
PR +	0	174 (53.21)
PR -	0	153 (46.79)
PR Status Missing	61 (100.00)	0
HER2 +	0	81 (24.77)
HER2 -	0	246 (75.23)
HER2 Status Missing	61 (100.00)	0
PAM50 Basal	15 (24.59)	42 (12.84)
PAM50 Her2	11 (18.03)	70 (21.41)
PAM50 Luminal A	13 (21.31)	103 (31.50)
PAM50 Luminal B	9 (14.75)	98 (29.97)
PAM50 Normal-like	13 (21.31)	14 (4.28)
Deaths	26 (42.62)	83 (25.38)
Mean Age	53.81	47.89

[a] *For Kao data, ER, PR, and HER2 status were inferred from expression data.*

Table S3: Distribution of Samples in Hatzis1 and Hatzis2 Breast Invasive Carcinoma Data

	Hatzis1 Count (%)	*Hatzis2 Count (%)*
Total Samples w/ pCR Data	306	182
Stage I	6 (1.96)	1 (.55)
Stage II	163 (53.27)	99 (54.40)
Stage III	135 (43.46)	82 (45.05)
Inflammatory	2 (0.65)	0
ER +	172 (56.21)	0
ER -	129 (42.16)	0
ER Status Indeterminate	4 (1.31)	0
ER Status Missing	1 (0.33)	182 (100.00)
PAM50 Basal	120 (39.22)	63 (34.62)
PAM50 Her2	20 (6.54)	16 (8.79)
PAM50 Luminal A	98 (32.03)	55 (30.22)
PAM50 Luminal B	43 (14.05)	32 (17.58)
PAM50 Normal-like	25 (8.17)	16 (8.79)
Metastases	64 (20.92)	42 (23.08)
pCR	86 (28.10)	51 (28.02)
Mean Age	50.16	48.85

Supplemental Results

Table S4: Features Selected by M2EFM

Data Type	Number of Features	Feature IDs
trans-m2eGenes	108	*ACAP1, ADAMDEC1, AFF3, AGR2, ANXA9, ASPN, BCL11A, C4A, CA12, CACNA2D2, CCL19, CCL5, CCR7, CD2, CD247, CD27, CD38, CD3D, CD3E, CD48, CD5, CD52, CD79A, CD79B, CD8A, CD96, CLSTN2, COL11A1, COL9A3, CORO1A, CXCL13, CXCL9, DACH1, DNAJC12, DNALI1, EGFR, EMILIN1, EN1, ERBB4, ESR1, FBP1, FOXA1, FOXC1, GABRP, GATA3, GFRA1, GREB1, GZMA, GZMB, GZMK, HLA-DQA1, IDO1, IGF2BP2, IL2RB, IL2RG, IRF4, ITK, KIAA1324, KRT16, LAMP3, LCK, LRRC15, LTB, LYZ, MAPT, MARCO, MLPH, MMP13, MS4A1, MSLN, MYB, NAT1, NKG7, PGR, PLA2G2D, POU2AF1, PRF1, PRKCB, PRLR, PSAT1, PTGDS, PTPRC, PTPRCAP, RARRES1, RET, ROPN1, S100A2, SCUBE2, SELL, SFRP1, SLAMF7, SLC16A6, SLC39A6, SLC44A4, SPDEF, SPOCK2, TBC1D9,*

		TFF1, TFF3, THSD4, TNFRSF17, TSPAN1, TTYH1, UBD, VGLL1, XBP1, ZAP70, ZMYND10
cis-m2eGenes	7	*BDH1, CCL14, DLG5, GSDMD, KIF5C, LILRB2, NCF4*
m2eQTLs	13	cg23576092, cg06423665, cg18033671, cg15114522, cg22895231, cg18013550, cg11335969, cg27331738, cg17010657, cg02076355, cg03801691, cg09227616, cg12707233

Table S5: Median C-index for Meta-Dimensional Overall Survival Models

Model	*Median C-index*	*p-values*[a]
Clin	.753	-
M2EFM Meth+Exp+Clin	.790	4.00e-03
M2EFM Exp+Clin	.787	5.00e-03
M2EFM-RF Meth+Exp+Clin[b]	.705	9.97e-01
Cox Meth+Exp+Clin	.728	8.24e-01
rorS+Clin	.775	1.80e-02
M2EFM Meth+Exp	.688	7.00e-03
M2EFM Exp	.682	1.10e-02
M2EFM-RF Meth+Exp[a]	.682	1.10e-02
Cox Meth+Exp	.662	5.70e-02
rorS	.636	2.13e-01

[a] p-values calculated from 1000 bootstraps of random gene sets equal in size to the relevant gene signature
[b] Alternative models that use Random Forest rather than ridge regression.

Table S6: Median C-index for Full Overall Survival Models

Model	*Median C-index*	*p-values*[a]
TCGA		
Clin	.746	-
M2EFM Exp+Clin	.759	1.00e-03
Cox Exp+Clin	.723	3.03e-01
rorS+Clin	.762	1.00e-03
NCA Exp+Clin	.757	2.00e-03
M2EFM Exp	.658	8.00e-03
Cox Exp	.660	8.00e-03
rorS	.631	9.70e-02
NCA Exp	.644	2.70e-02
Terunuma		

Clin	.653	-
M2EFM Exp+Clin	.721	1.12e-01
Cox Exp+Clin	.720	1.32e-01
rorS+Clin	.678	7.44e-01
NCA Exp+Clin	.652	9.54e-01
M2EFM Exp	.632	2.89e-01
Cox Exp	.661	8.00e-02
rorS	.601	6.02e-01
NCA Exp	.563	8.66e-01
Kao		
Clin	.637	-
M2EFM Exp+Clin	.697	2.57e-01
Cox Exp+Clin	.726	4.00e-03
rorS+Clin	.666	9.09e-01
NCA Exp+Clin	.670	8.62e-01
M2EFM Exp	.684	7.20e-02
Cox Exp	.725	0.00
rorS	.632	7.15e-01
NCA Exp	.621	8.41e-01

[a] p-values calculated from 1000 bootstraps of random gene sets equal in size to the relevant gene signature

Table S7: Hazard Ratios for Final Overall Survival Model

	HR	*95% CI*	*p-value*
Molecular Risk	3.32	[2.58, 4.28]	< 2.00e-16
Stage II	1.50	[0.86, 2.61]	1.60e-01
Stage III	3.52	[1.96, 6.33]	2.59e-05
Stage IV	9.52	[4.55, 19.92]	2.21e-09
Age	1.04	[1.02, 1.05]	6.27e-07

Table S8: Median C-index for DRFS Survival Models

Model	*Median C-index*	*p-values*[a]
Hatzis1		
Clin	.646	-
M2EFM Exp+Clin	.722	3.50e-02
Cox Exp+Clin	.701	3.17e-01
rorS+Clin	.707	2.07e-01
M2EFM Exp	.705	2.10e-02
Cox Exp	.690	1.08e-01
rorS	.663	5.79e-01

Hatzis2		
Clin	.578	-
M2EFM Exp+Clin	.752	0.00
Cox Exp+Clin	.735	3.00e-03
rorS+Clin	.658	9.62e-01
M2EFM Exp	.741	4.00e-03
Cox Exp	.729	3.30e-02
rorS	.634	9.98e-01

[a] p-values calculated from 1000 bootstraps of random gene sets equal in size to the relevant gene signature

Table S9: Hazard Ratios for Final Distant Recurrence-Free Survival Model

	HR	*95% CI*	*p-value*
Molecular Risk	4.07	[2.82, 5.89]	8.69e-14
Stage III	1.84	[1.10, 3.08]	2.12e-02
Age	1.01	[0.98, 1.03]	6.65e-01

Table S10: Median C-index for pCR Models

Model	*Median C-index*	*p-values[a]*
Hatzis1		
Clin	.588	-
M2EFM Exp+Clin	.739	4.34e-01
Logistic Exp+Clin	.767	2.20e-02
M2EFM Exp	.733	1.00e-02
Logistic Exp	.763	0.00
Hatzis2		
Clin	.507	-
M2EFM Exp+Clin	.716	7.30e-02
Logistic Exp+Clin	.707	1.49e-01
M2EFM Exp	.727	4.90e-02
Logistic Exp	.709	1.90e-01

[a] p-values calculated from 1000 bootstraps of random gene sets equal in size to the relevant gene signature

Table S11: Odds Ratios for Final Pathologic Complete Response Model

	OR	*95% CI*	*p-value*
Molecular Risk	4.85e-04	[5.91e-05, 3.36e-03]	1.12e-13
Stage III	4.78e-01	[2.57e-01, 8.66e-01]	1.68e-02
Age	9.88e-01	[9.60e-01, 1.02e+00]	4.09e-01

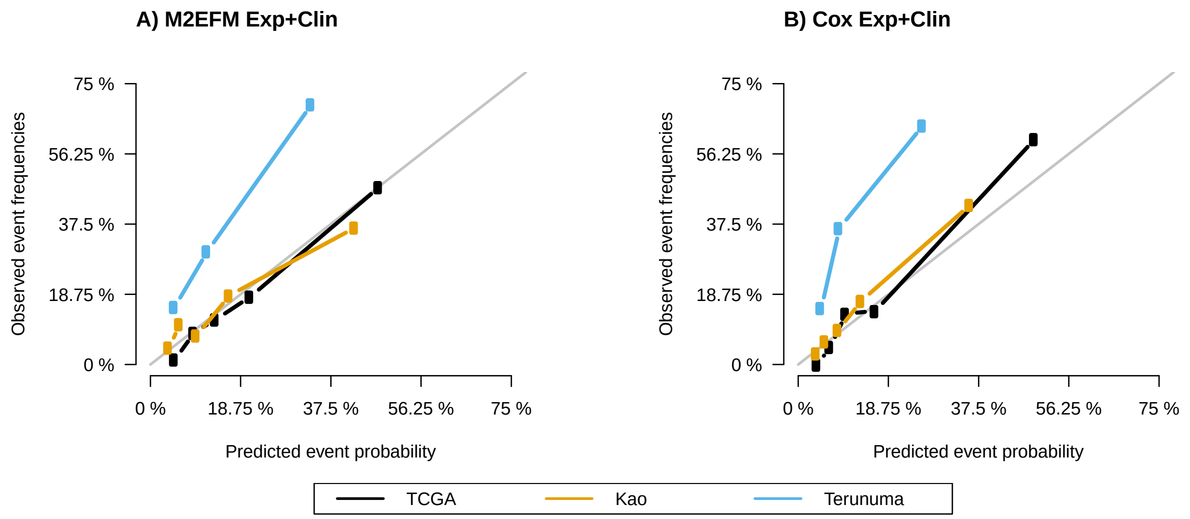

Figure S1: Calibration curves at 5 years for overall survival outcome models for M2EFM Exp+Clin (A) and Cox Exp+Clin (B).

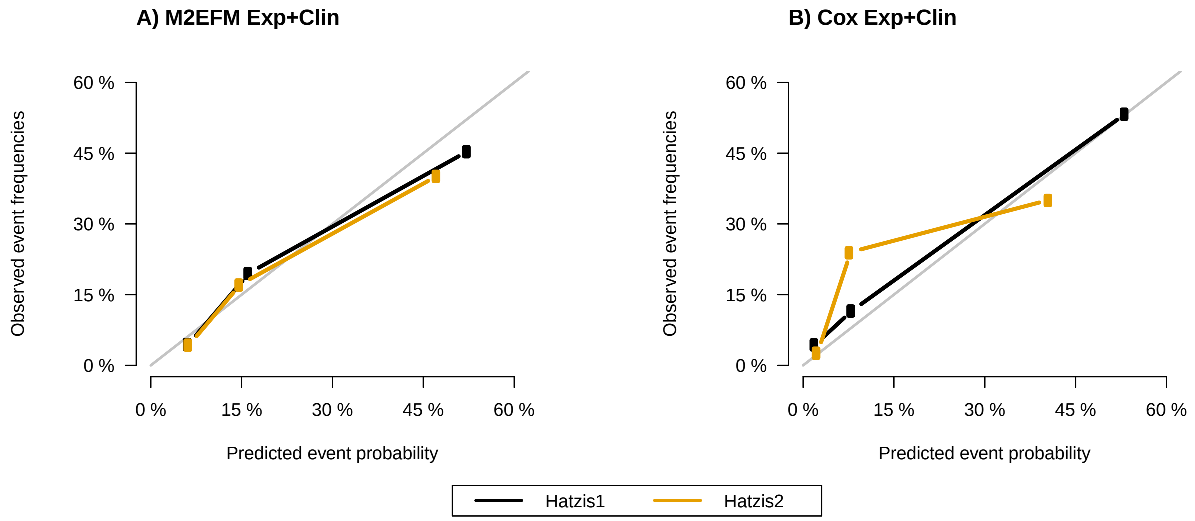

Figure S2: Calibration curves at 3 years for distant recurrence free survival outcome models for M2EFM Exp+Clin (A) and Cox Exp+Clin (B).

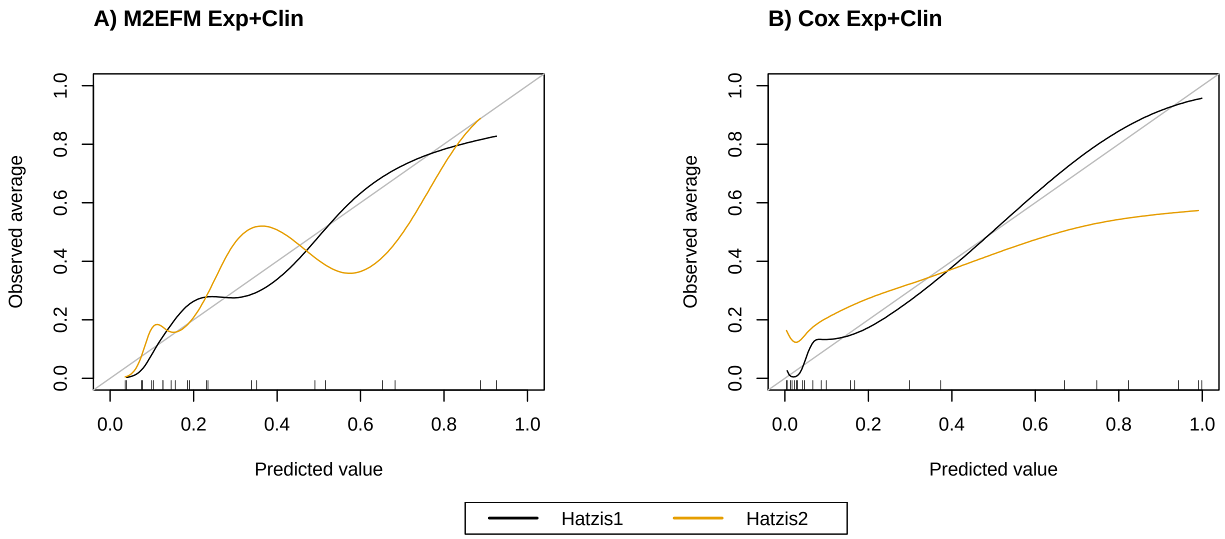

Figure S3: Calibration curves for pathologic complete response models for M2EFM Exp+Clin (A) and Cox Exp+Clin(B).

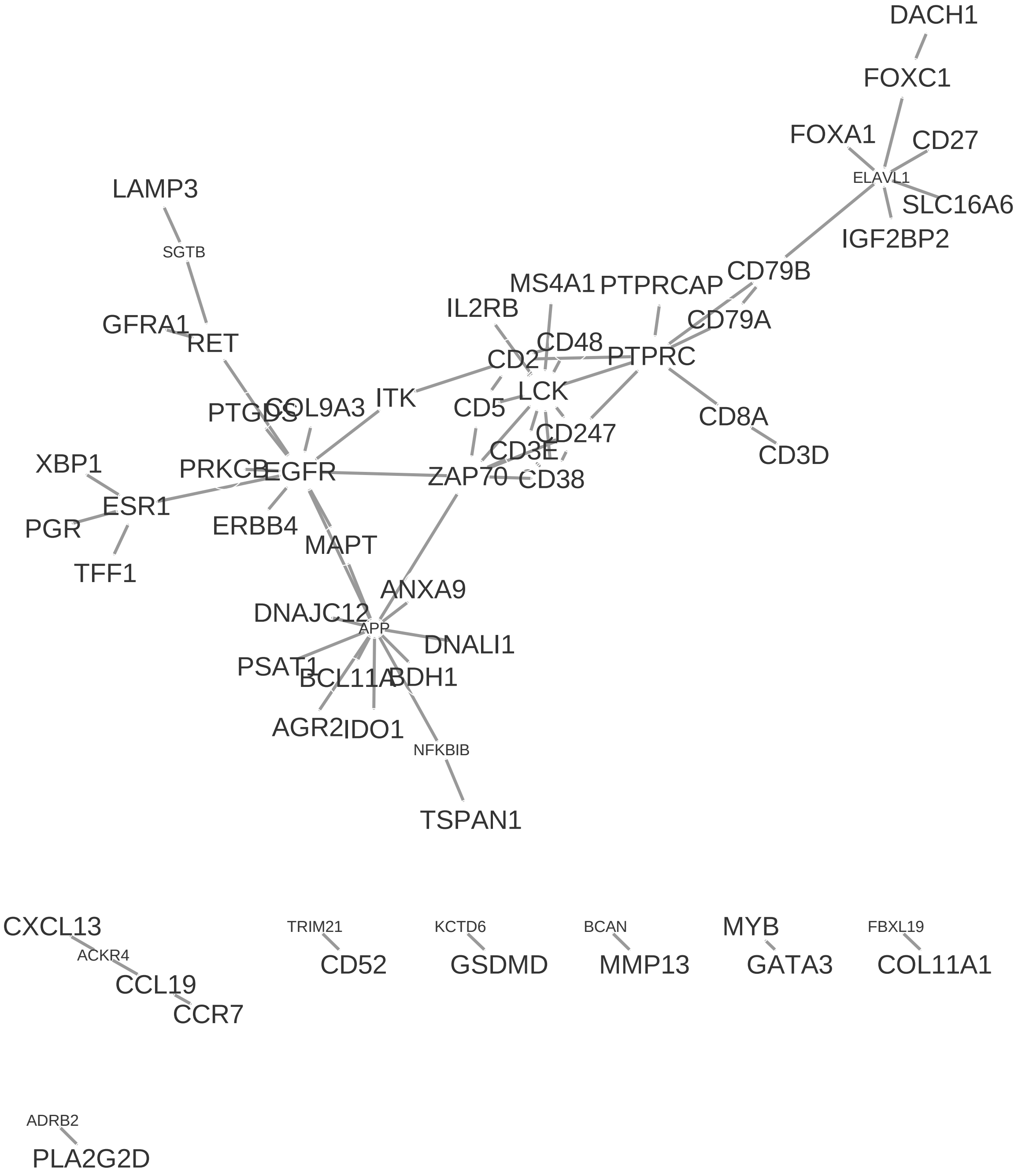

Figure S4: Sub-network of protein-protein interactions from WebGestalt, enriched with m2eGenes, which are shown as larger circles.

References

1. Ritchie MD, Holzinger ER, Li R, Pendergrass SA, Kim D. Methods of integrating data to uncover genotype–phenotype interactions. *Nat Rev Genet*. **2015**;16:85–97.
2. Teschendorff AE, Gao Y, Jones A, Ruebner M, Beckmann MW, Wachter DL, et al. DNA

methylation outliers in normal breast tissue identify field defects that are enriched in cancer. *Nat Commun*. **2016**;7:10478.

3. Shabalin A a. Matrix eQTL: ultra fast eQTL analysis via large matrix operations. *Bioinformatics*. **2012**;28:1353–8.
4. Hoerl AE, Kennard RW. Ridge Regression: Biased Estimation for Nonorthogonal Problems. *Technometrics*. **1970**;12:55–67.
5. Hatzis C, Pusztai L, Valero V, Booser DJ, Esserman L, Lluch A, et al. A genomic predictor of response and survival following taxane-anthracycline chemotherapy for invasive breast cancer. *Jama*. **2011**;305:1873–81.
